# Edge and Fog Computing Platform for Data Fusion of Complex Heterogeneous Sensors

**DOI:** 10.3390/s18113630

**Published:** 2018-10-25

**Authors:** Gabriel Mujica, Roberto Rodriguez-Zurrunero, Mark Richard Wilby, Jorge Portilla, Ana Belén Rodríguez González, Alvaro Araujo, Teresa Riesgo, Juan José Vinagre Díaz

**Affiliations:** 1 Centro de Electrónica Industrial, Universidad Politécnica de Madrid, José Gutiérrez Abascal 2, 28006 Madrid, Spain; jorge.portilla@upm.es (J.P.); teresa.riesgo@upm.es (T.R.); 2 B105 Electronic Systems Lab, ETSI Telecomunicación, Universidad Politécnica de Madrid, Avenida Complutense 30, 28040 Madrid, Spain; r.rodriguezz@b105.upm.es (R.R.-Z.); araujo@b105.upm.es (A.A.); 3 Group Biometry, Biosignals, Security, and Smart Mobility, Universidad Politécnica de Madrid, Avenida Complutense 30, 28040 Madrid, Spain; mrwilby@etsit.upm.es (M.R.W.); abrodriguez@etsit.upm.es (A.B.R.G.); jjvdiaz@etsit.upm.es (J.J.V.D.)

**Keywords:** edge computing, fog computing, data fusion, multi-sensor systems, complex sensors

## Abstract

The explosion of the Internet of Things has dramatically increased the data load on networks that cannot indefinitely increment their capacity to support these new services. Edge computing is a viable approach to fuse and process data on sensor platforms so that information can be created locally. However, the integration of complex heterogeneous sensors producing a great amount of diverse data opens new challenges to be faced. Rather than generating usable data straight away, complex sensors demand prior calculations to supply meaningful information. In addition, the integration of complex sensors in real applications requires a coordinated development from hardware and software teams that need a common framework to reduce development times. In this work, we present an edge and fog computing platform capable of providing seamless integration of complex sensors, with the implementation of an efficient data fusion strategy. It uses a symbiotic hardware/software design approach based on a novel messaging system running on a modular hardware platform. We have applied this platform to integrate Bluetooth vehicle identifiers and radar counters in a specific mobility use case, which exhibits an effective end-to-end integration using the proposed solution.

## 1. Introduction

Wireless Sensor Networks (WSNs) is not a new idea, they have been around for more than 40 years [1]. Nevertheless, their architecture on implementation is still in progress. Today, WSNs have evolved to incorporate bigger heterogeneous networked systems, ranging from the cloud to the extreme edge. The proliferation of sensors and related devices has been dramatic, embodied in the now ubiquitous Internet of Things (IoT). This has led to the boundary between the physical world and the digital being constantly eroded by the IoT, which complicates the nature of what information is being generated and where it is needed. However, it is only the early stages of a revolution in access to real-world information. We are currently dealing with not only the incremental evolution of traditional sensors in terms of accuracy, latency (communications), etc., but also a fundamental change in the nature of the capabilities of sensors, as well as the growing realization that the significance of the information generated by these sensors depends on the physical observations that may be monitored by other sensors, i.e., information fusion.

These changes can be interpreted in terms of the different IoT levels, in what has been called the continuum cloud-to-things [2]. The bottom layer is located at the edge (where data is created.) Connecting these edges is the computation plan that provides significance to the data collected. Centralised computation leads to massive data uploading to the cloud and latency issues, which can be alleviated by bringing decision capabilities to the things [3]. The ability to compute allows the dynamic development of edge devices where operations can be evolved bringing functional changes to the information that can be retrieved [4]. Specifically, localised processing supports the transformation of the collected data into forms more appropriate to the actual monitoring task. Processing raw data typically provides a compression function as well as making the collected data easier to interpret and treat. Additional advantages include reduction in data center cost, decrease of network traffic, scalability increase and simpler IoT devices. However, bringing more computing close to the physical entities introduces new challenges in terms of allocation of resources and real time needs. These challenges are related to the computation offloading among distributed edge devices to avoid hot spots and bottlenecks, distribution of tasks between cloud and edge, communication issues derived from the mobility and security have to be re-evaluated, to name just a few.

In practical terms this revolution has been precipitated by the introduction of cheap single-board computers (SBC), which has altered the nature and complexity of sensors and the ability to deploy them. We are no longer restricted to simple devices capable of collecting a direct measure of certain variables from the environment, e.g., thermometers or light sensors. We are now open to the relatively easy development of other systems that need some kind of processing to extract a first level of information from the raw data they retrieve. It should be noted that the type of processing can change the nature of the provided information received from a physical sensor. Specifically a data stream coming from a camera can be transformed into an arbitrary number of functions (text reading, object tracking, etc.), so a single real sensor can in fact look like, or contribute to, a multitude of different apparent sensors. We use the generic term *complex sensors* to refer to devices in this class of sensors.

In addition, the ease of fabrication of new types of “Things”, noted as an advantage, is also problematic. The reduction in complexity gained by having local computation has been exacerbated by the introduction of cheap standard components which can be bolted together to produce novel systems making the development of a new type of sensor considerably easier. This means today, the introduction of a new type of sensor is simply one element of a project, rather than being the project itself. It is not fashionable, but this ability to add new physical capabilities via the introduction of new customised hardware and how they can be made to interact with and extend existing systems is a day to day practical issue. This issue has resulted in the need for a methodology to facilitate the integration of hardware and software development.

### 1.1. Motivation

The work we present in this paper grew out of several collaborative projects between the three research groups that form the team. In these projects we developed and deployed a range of complex sensors, where we repetitively faced a common set of integration issues. Invariably this required rapid re-prototyping of hardware systems and going beyond integration of heterogeneous devices requiring a simple and reliable form of communication between software and hardware teams.

Furthermore, in most cases, complex sensors generated huge amounts of raw data that had to be processed locally in order to avoid dramatic decreases in the overall performance of the system.

No generic platforms were found that could provide us with a common framework that could facilitate the integration and development of sensors and applications across not just software boundaries, but also hardware interfaces. Consequently, we needed to produce a hardware and software integration that can extract information at the edge to let developers scale both devices and applications.

### 1.2. Contributions

In order to fulfil the previously stated need, we built a complete information framework based on a novel messaging system shared by both the software application modules and a hardware integration platform, namely the Cookie [5]. This resulting common development framework is the main contribution of this paper, achieving a set of improvements:

1. It eases the integration of complex sensors into new or existing networks and applications.
2. It extends the concept of the *sensor* itself, evolving towards a *source of data* so that anything generating data (simple and complex sensors, databases, open-data platforms, processing modules...) could be seamlessly treated and fused in order to develop growing applications.
3. It simplifies the interaction between hardware and software developers. It provides a foundation for the coordination of teams jointly working on new sensors and/or applications. Although we are not presenting quantitative comparisons, we believe this leads to both a time and cost reduction in development.
4. Finally, in order to illustrate these benefits, we have constructed an integrated traffic monitoring sensor that provides both vehicle counting and identification. Fusing the complementary data this sensor produces, we can obtain extended traffic information, like weighted travel times, that none of the original sensors could provide on their own.

## 2. Related Work

### 2.1. Edge Computing

Debates regarding which is the best place to situate computing and storage resources have been going on since the first computers were networked. On the other side, the answer to such questions will always be the same: it depends on what you are trying to do. Cloud computing [6] contributes most in its attempt to provide resources in a transparent and usable form, where the application can adapt the resource to its requirement, rather than the other way around. As a complementary perspective, *edge computing* [7] focuses on the periphery of the network.

WSNs can use any of these basic approaches. However, the networked nature of the IoT problem, the increased use of SBCs, improvements in data processing technologies, and the reduction in price of sensor devices all lead quite obviously to computational resources close to the sensors and hence to edge computing, and more specifically *fog computing* design methodologies. Fog computing was defined as a way to bring computing and storage resources closer to the data generation. This is not in essence very different from the definition of the edge [8]. Even some researchers do not distinguish between fog and edge. Their main difference is on the dimension of the hardware elements and the processing and storage capabilities resident in the fog and the edge. One aspect that is a key distinction among them is that fog does need the cloud to exist. The type of devices that are traditionally connected with the fog are network routers, switches and embedded servers, although the heterogeneity and the effect of SBCs is present in this layer as well.

In fact, edge computing has been shown to be essential to the evolution of IoT [9], but where it is generally seen as a software strategy, we regard it as a solution to a hybrid world of innovation including ongoing hardware developments. As it has been mentioned previously, edge computing can help to solve some inherent problems of the IoT associated with the massive generation of data. The devices located in this level of the IoT may be very different to each other, and usually this brings other distinction in two layers: the edge and the extreme edge. The main difference among both layers resides in the nature of the devices that are measuring and processing data from the physical world, form the point of view of power consumption, processing capabilities, and communication systems.

The edge now contains gateways acting as bridges onto specific sensor systems as well as hardware embedded devices SBCs all the way through to WSNs, e.g., TelosB motes or similar ones are found [10]. Many of these devices are powered from a mains supply because they have to operate for months to years and when there is significant computation, there is sizeable power consumption. In many use cases where mobility is not a factor, power availability is not as uncommon as people first expect. All of these devices need batteries when power is not available as although consumption can be made low (in the order of milliwatts), it is still significant. Lifetimes of months are difficult to obtain, when the processing is more intensive than just monitoring and reporting intermittently.

The extreme edge heralds a new generation of highly resource-limited devices with virtually no memory, processing capabilities, and local energy store. To operate such systems we require energy harvesting techniques, to get what has been called zero or almost zero-power sensors. Some good examples of devices in this layer are [11,12]. Such devices are crucial for a future realistic implementation of the IoT paradigm and will only be able to operate by interworking with edge devices described previously. The development model defined here is ideal for supporting such systems.

### 2.2. Hardware Integration Platforms

The explosion of the IoT has brought an important evolution of hardware and software technologies on the edge to cope with the limitations and the intrinsic characteristics of real in-field sensor deployments (and even setting novel requirements according to the evolved nature of the present and future networks). This fact also implies challenges regarding how to properly handle the diversity and complexity of the sensing technologies to be deployed. In this way, an end-to-end platform takes advantage of a modular architecture to allow fast integration of sensing, processing, and communication capabilities on the edge, based on a common hardware definition to fully support the concept of heterogeneous complex sensors.

The Cookies platform [13] was conceived to guarantee a flexible and seamless integration of different technologies thanks to the modular nature of the architecture, where individual elements that compose the sensor node can be replaced or updated without altering the overall structure of the platform. Consequently, it fosters the re-usability of hardware components and it is also designed for the re-use of its software components. The modularity of the Cookies empowers the concept of integrating heterogeneous complex sensors on the edge by providing a well-defined hardware framework and basic rules for the implementation of different and multiple sensing capabilities.

Although such a modularity allows including a different number of layers into the hardware stack, the basic composition of a Cookie node features 4 main layers that correspond to the baseline functionalities of a WSN and IoT edge device. First, the communication layer where the low-power networking technology is integrated so as to supply the sensor node with wireless connectivity with the surrounding edge elements and the fog layer (direct or indirect according to the type of topology to be adopted). The intrinsic nature of the Cookie platform as a fast-prototyping architecture has led to integrate different wireless communication standards, including the well-established WSN IEEE 802.15.4 and ZigBee stacks, and also more resent specifications such as the Internet Protocol version six (IPv6) over Low-power Wireless Personal Area Networks (6LowPAN) and the Long Range Low Power, Wide Area (LoRa) network protocols.

Second, the sensor layer, in which the physical interaction with the target environment is performed by integrating the sensing technologies and, in particular, the complex sensors that operate within the proposed heterogeneous edge nodes. The Cookie architecture has allowed the implementation of different sensing capabilities during the development and deployment of real WSNs in real application scenarios, encompassing carbon dioxide, air alkalinity and pH sensors for environmental and industrial monitoring.

Third, the processing layer, which represents the core of the edge node and it is in charge of performing the management of the overall sensor device, ranging from basic sensing retrieval and raw data transmission to the base-station, up to further local processing and data fusion of complex sensors in accordance with the IoT application requirements. Indeed, such requirements will also define the specification of the type of processing elements to be integrated. In general terms, the main Cookie processing layer is composed of 8051-based microcontrollers (as described subsequently in the use case section) particularly those designed for low-power and ultra-low-power purposes. In addition, other processing architectures have been used, comprising the very well-known MSP430FG439 microcontroller from Texas Instruments (Dallas, TX, USA) [14] and ARM-based processors such as the ATSAMD21 from Microchip (Chandler, AZ, USA) [15] that features a Cortex-M0+-based 256 KB flash low-power microcontroller.

Finally the power supply layer, which provides the different voltage levels to the rest of the hardware elements in the Cookie node. Depending on the input power source (typically a battery), this layer is designed to satisfy the power requirements of the processing, communication and sensing technologies in such a way that the stability and long-term operability of the overall node is assured. This means that additional power adaptation apart from typical linear regulators (for instance, the inclusion of a power management unit) can be performed in this layer. The general architecture of the Cookie nodes establishes standard voltage levels that shall be taken into account when designing a new modular layer, although this does not limit the specification of additional reference values or even the implementation of further dedicated power transformations into the design of a custom layer.

As it can be seen, the Cookie node serves as a technology gathering platform to speed up the research and implementation of particular functionalities by just replacing or combining several pieces of hardware layers into the sensor nodes. This means that new prototypes can be integrated in combination with already well-established layers, so as to facilitate the experimentation of new standards and methods under the umbrella of a supportive and stable platform.

**Figure 1:**
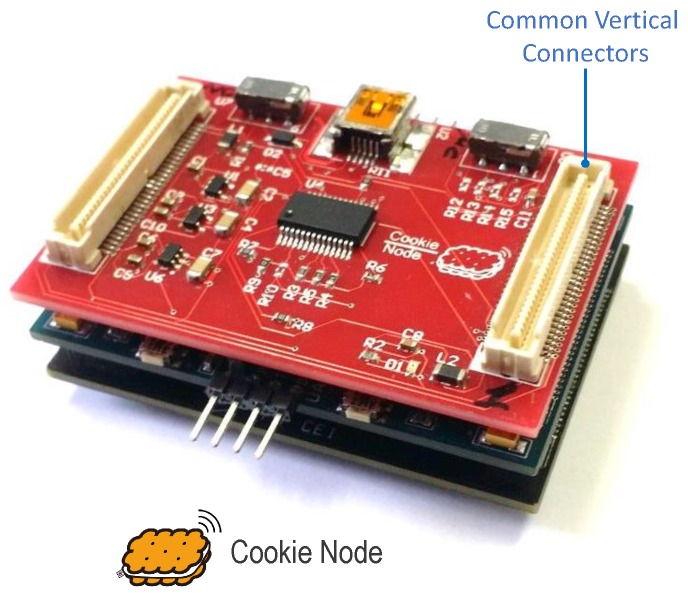
Modular Hardware platform: Cookie node.

As shown in Figure 1, the general architecture of the modular platform is primarily composed of a set of common interfaces that outline a three-side hardware integration: power supply distribution, analog signal provision and digital interfaces. This sensor platform provides support for a diversity of different communication interfaces through the availability of the vertical connectors highlighted in Figure 1. In particular, three serial interfaces are dedicated to communicating with the sensor layers, i.e., Serial Peripheral Interface (SPI), Inter-Integrated Circuit (I2C) and Universal Asynchronous Receiver-Transmitter (UART).

So in general the design and integration of heterogeneous sensor layers within the Cookies shall only consider three main aspects at hardware level: the disposition of the vertical connectors that will ultimately provide the desired interface to the complex sensors; the compliance with the power supply, i.e., the voltage signal distribution and the power consumption margins, which does not limit the inclusion of any particular adaptation into the sensor layer design itself to assure its adequate behavior and the use of any of the provided serial buses to properly exchange the raw information with the embedded sensor manager.

**Figure 2:**
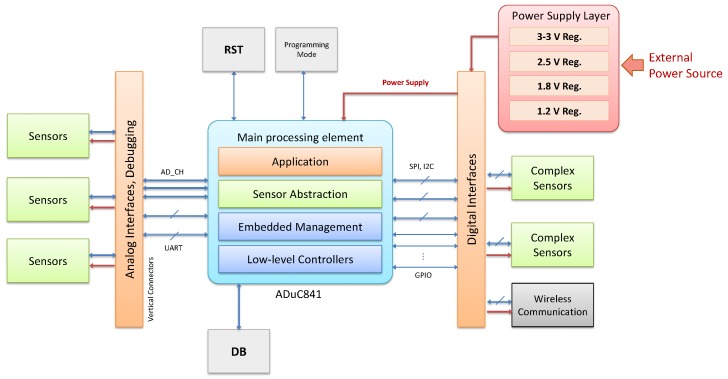
General view of the hardware architecture of the Cookies.

The processing layer of the Cookie node represents the basic core element to gather and generate information locally from the connected sensors, and thus it is in charge of managing the initialization, configuration and direct communication with those sensing elements. As for the rest of the Cookie layers (i.e., sensing, communication and power supply), the processing layer can be indeed adapted to the particular requirements or constraints of the target application context. Anyway, as shown in Figure 2, the general structure of the typical processing layer of a Cookie node is composed of a main low-power 8051-based microcontroller upon which the embedded management system for the complex heterogeneous sensors is implemented, while the possibility of a secondary co-processing element based on a Field-Programmable Gate Array (FPGA) is contemplated (in case higher performance is needed).

Figure 2 depicts the general structure of the Cookie node from the perspective of the processing layer. In orange, the vertical connectors that provide a common interconnection among the modular layers and allow the data and power signals exchange throughout the whole platform (in blue and red respectively). Green boxes represent the integration of different independent complex sensor layers considering the proposed digital interfaces, although the use of analog signals is not restricted; the gray box illustrates the communication layer. In the center of the diagram, we show the main processing element that supports the implementation of the complex sensor management system. It relies on an embedded software that follows the layer-based architecture of the modular platform, so that the processing and data fusion of the complex sensors is developed upon the platform abstraction API, the software controllers, and the Cookie support libraries.

As shown in Figure 2, the general structure of the typical processing layer of a Cookie node is composed of a main low-power 8051-based microcontroller upon which the embedded management system for the complex heterogeneous sensors is implemented, while the possibility of a secondary co-processing element based on an FPGA is contemplated (in case higher performance is needed).

### 2.3. Complex Sensors in Traffic Monitoring

Traffic monitoring has traditionally used a whole set of technologies in order to monitor vehicles and collect usable data representing traffic states. All these technologies can be classified into two main groups: (i) counting and (ii) Automatic Vehicle Identification (AVI). Technologies like inductive loops and radars, falling on the former category, focus on calculating variables related to detecting a vehicle passing by a specific point on the road; primarily intensity (representing the number of vehicles per unit time), occupancy (percentage of occupied road segment), and velocity. The latter provide a unique identifier of vehicles, thus allowing the calculation of variables like travel times and origin-destination matrices.

Among counters, Doppler radar technology [16] has been long used in traffic sensing applications. These radars transmit a microwave signal to a desired target in order to obtain its velocity data from the altered frequency of the returned signal. Therefore, traffic counting can be achieved by processing radar sensor data which provides velocity of the vehicles in its range.

A radar transceiver generates the high-frequency signals that are transmitted through the air. When a moving target is in the radar range, a frequency-deviated signal is reflected. This reflected signal is received by the radar sensor and is used together with the transmitting signal as input of a frequency mixer. The output of this mixer is a low-frequency signal whose main components have the frequency deviation caused by moving targets. Finally, I and Q components are extracted providing phase information of the received signal.

A radar sensor integrates a hardware module and a software processing unit. The signal generation, as well as the demodulation of returned signals, amplification and filtering is done by the hardware module. Then, the returned signals are digitalized in order to be processed by the software unit which provides counting information. First, digital signal filtering is performed to reduce undesired frequencies and a windowing function is applied. Then, a Fast Fourier Transform (FFT) is performed to obtain frequency information from I and Q signals. The velocity of a moving target is directly related to the frequency components of these transformed signals and the direction of movement is inferred from the sign (positive or negative.) Finally, the velocity data obtained is used to achieve vehicle counting. A vehicle count is added each time a non-zero velocity is detected and then the system waits for the next counting for a configurable time to prevent false positives. According to these features a radar counter is considered to be a complex sensor given that it needs to perform complex operations to generate a first level of usable data.

On the other hand, Bluetooth (BT) vehicle identifiers are one of the latest contributions to AVI technology [17]. A BT chip forms the basis of the vehicle identification sensor. As described in [18], this chip is programmed to run in a scanning mode, namely the *inquiry process* included in the standard. Under this operation, the BT chip scans each of the 32 transmission channels available in order to detect devices in the coverage area. Each BT device emits its unique identifier, the MAC (Medium Access Code) address, to discover other BT devices in their vicinity. In addition, in systems compatible with version 3 or lower, which is still very common in in-vehicle systems, they also transmit a second identifier, the DIAC (Dedicated Inquiry Access Code), a 24-bit code including two classes: the *device class*, which describes the type of BT chip; and the *service class*, which refers to the service it provides. We use the DIAC to keep only those BT devices that are directly related to a vehicle, i.e., the hands-free appliances embedded. Thus, in summary, our BT sensor identifier monitors the surrounding area, detects the identifiers of the BT devices traversing it, filters out those identifiers that do not correspond to vehicles, and generate a data output that includes: the sensor identifier, MAC, DIAC, and time stamp.

Although data fusion from different vehicle monitoring technologies would enable a deeper understanding on how traffic behaves [19], there is currently no solution that optimally fuses data from these two types of sensors, counters and identifiers. In consequence, we can also access partial information about traffic. For example, we are not able to know precisely the percentage of the flow of vehicles approaching a crossroad (intensity) that actually turned right, left, or continued straight forward (origin-destination matrices).

## 3. Integration Strategy Based on Symbiotic Hardware/Software Design

Edge and fog computing bring processing capabilities to the proximity of sensors, thus avoiding network overload and other subsequent inefficiencies. Complex sensors specifically require this functionality in order to provide a first level of useful data that do not come directly from the raw data they collect from the environment. However, edge and fog computing themselves are not capable of providing an overall solution to data fusion of complex sensors. Data fusion imposes restrictions to both hardware and software design of sensors and systems. These restrictions limit the flexibility of hardware and software developers to optimize the performance of their corresponding target deliverables and force them to work tightly together in order to comply to the set of double fold requirements. This issue is aggravated in the presence of complex sensors given the prior processing they demand.

Consequently, we must provide hardware and software developers of sensors and applications with a framework that allows them to work within their particular scope and abstracting the remaining fields of the overall objective. Our solution to this issue is to define a software abstraction that mirrors the physical reality of the sensor systems and define sensors in term of a message definition process.

### 3.1. Messaging System Architecture

Our message name space is tied to sensors, so each sensor has a *type* (name space) that defines it. The sensor then is defined by the messages it produces and consumes making this a Service-Orientated Architecture (SOA), with the services placed at the edge of the network. This means designing a sensor is simply designing these messages and their interactions. This process is automatically forced into a shared ground and more importantly a common language between software and hardware developers. The resulting message design provides a unique framework for both hardware and software development. Additionally, it defines an efficient process to deal with design life-cycle: initial design limitations, upgrades, and extensions.

The messages can take a multitude of different forms. Most commonly we employ a standard JavaScript Object Notation (JSON) representation, used predominately on the network side, and a compact serialized form frequently used for communication across serial ports. Nevertheless, there is no restriction to the forms that can be constructed. Regardless of its form chosen, the messages can trivially be encapsulated in any transport medium and no knowledge of the message structure, or sensor type, unless the system at this point needs to interact with the sensor. This representational flexibility also makes it easy to integrate existing, or proprietary sensor technologies into the framework.

The ability to transform the message representation to suit the immediate task at hand has many advantages in all parts of the network. However, it is important to realize that any representation still describes the message and it does not need transforming, nor does the transport technology need to know anything about it. We have developed a middle-ware technology which builds on a broker-less messaging platform that is optimized for the use inside the network to coordinate large scale sensor deployments. However, this is not the focus of our current argument, as any transport strategy can benefit from the use of this approach at the gateway/sensor interface. There are many technologies that can be used at the transport level, traditionally Message Queuing Telemetry Transport (MQTT) [20], or Advanced Message Queuing Protocol (AMQP) [21], or more modern approaches such as [22].

The abstract representation of a message is more than just a schema. It provides precisely a description of what data is contained in the message. Not for a given syntax, but for any representation imaginable. We started out by having a YAML Ain’t Markup Language (YAML)-based definition language for messages, but over time we realized any given representation was sufficient to act as the description. JSON’s readability made it ideal for this purpose and using it for example messages has effectively eliminated the need for a definition language, or formal schema representation. The fact that in end applications a JSON format makes the message completely human readable can be a major advantage. However, if a particular application requires it, a more compact representation could just as easily be used.

Any combination of hardware/software developers can design the message sequence. In the most common scenario, the hardware engineers initiate the design and then (possibly, but not always) software engineers review and extend it to match requirements from the network side. Once an agreed document is in place the two sides can start to work independently. On the hardware side, messages are asynchronous, so there is no restriction to lock input messages to output messages. In theory hardware engineers have to be aware of the physical level protocol between the gateway and the physical device, but in reality this is simply replaced by a library call to sending and receiving messages. The software engineers simply need to provide handlers for the processing of the messages. Messages only need to be processed when details of the sensors function are required. Messages are simply encapsulated payloads, although within our framework they do have some very simple structure to allow “in transit” processing and to optimize multiplexing processes. Synchronous messaging is handled automatically within our messaging system, but this is a convenience rather than a necessity.

The fact that libraries designed to parse and manage the messages are constructed as a by-product, is the principle gain for the hardware/embedded systems developer. Very quickly, they no longer have to worry about the message interactions between the gateway and the real sensor and focus their activities on the operation of the sensor.

**Figure 3:**
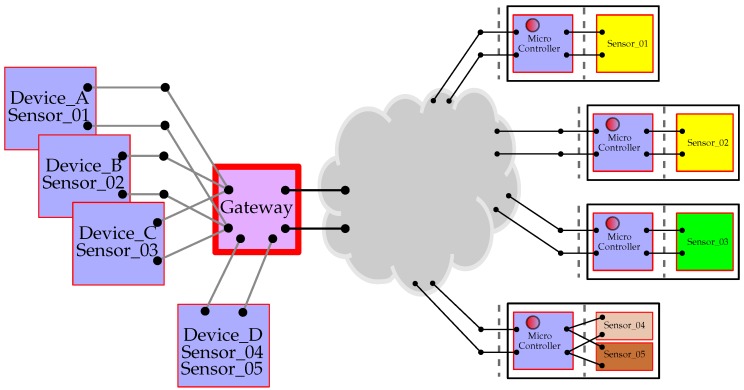
Abstract structure of an operational gateway element. The actual physical distribution of gateway, device nodes and attached sensors is mirrored in a software model that shadows the same distribution. It is not necessary for the software shadow to match exactly the physical deployment, which is where local data fusion can begin.

The standardization of all sensor processes is completed by a common conceptual framework, where we decompose the edge system into 3 components: *sensor*, *device*, and *gateway*. Figure 3 shows this architecture. As we can observe, each physically connected equipment has its corresponding software shadow that acts as the protocol bridge to the real physical system.

### 3.2. Sensor

The sensor provides a software shadow of the real sensor. Specifically, the sensor in the framework is a software representation of a real sensor, or group of real sensors. Real sensors are physically connected in some way to the system, which also has its own software correspondence in the *Device* and *Gateway*. The real sensor responds to message requests in some asynchronous way and generates response messages accordingly. Its software shadow handles the messages that come from and go to the real sensor. To do this the architecture routes messages to the gateway. Those messages that are not destined for other network elements or the gateway itself are routed through to the corresponding device controllers. The device controller is also a basic router whose job is to route the message to the appropriate sensor element.

Based on this approach, the sensor, in terms of the framework, is defined by the messages it can receive and send. A message is described in detail later, but it is basically a complex collection of data elements. The software architecture of the shadow sensor takes the form:

- **Sensor body.** This is the core element and contains the basic operational logic of sensors. It handles the representation model of the sensor, holds copies of, or links to, all the semi-persistent data, and acts as a container for the other two components.
- **Message Processor.** This processes the incoming messages and performs any local action necessary before the message is transferred to the real sensor. It also converts any message that is in the general JSON-based format to the serial format, or vice versa, if necessary.
- **Message Handlers.** There can be several of these to process the messages generated by the sensor and pass them (or a processed version of them) to the appropriate network connection. It is typically in one of these handlers that the generated message can be transformed into a different representation. If it is not required such a handler is not included.

According to this architecture, a sensor is described as a software object with its own definition as we present in Listing 1. We previously stated that both the real sensor and its software shadow have a type, which is defined in terms of the messages it can send and receive. The Message Processor and the Message Handlers are associated with this type.

**Listing 1:**
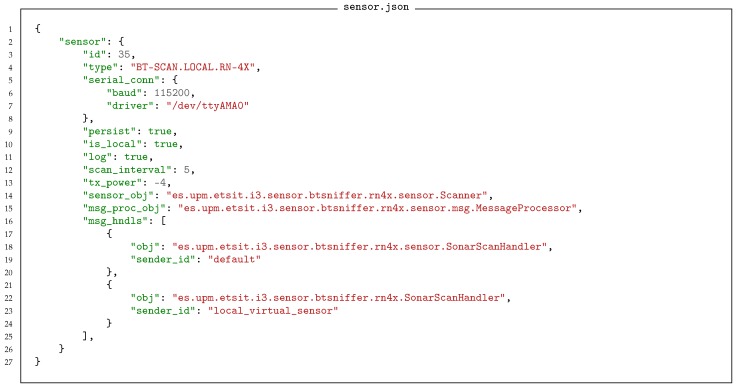
An example of a sensor definition. It is given a type and defined in terms of core element, message processor, and message handlers.

Making use of the Message Processor on the sensor shadow, any processing module or application can communicate with any real sensor connected to the platform both in an asynchronous or a synchronous way. Even though the real sensors only ever implements asynchronous messaging. Once a message is received on the shadow sensor it can be sent directly to the network through the Device and Gateway or processed locally by means of a Message Handler. A Message Handler provides specific functionality to the sensors, which is particularly relevant when dealing with complex sensors.

It should be noted that the sensor described in Listing 1 is a locally defined sensor. Specifically, it is a software sensor designed to run on the local hardware. To that end the definition contains device specific information (communications port, module transmission power etc.). Such software sensors are typically defined in the gateway specification file, Section 3.4, as they typically only exist at runtime and the gateway needs to know that it has to create them. Alternatively, a physical device can implement the sensor. In this case the sensor body is a simple ghost used to route messages to and from the sensor, whilst the handlers are the same. The specific configuration of the sensor is defined via a sensor factory which instantiates the shadow sensor based on the received sensor type information. Both the software defined sensor and the physical sensor define exactly the same message structures, so the operation of both seem identical to the system. Note, that in the system designed in this deployment the BT scanner sensor is not implemented as a local device, but as a Cookie device.

### 3.3. Device

The device represents the controlling element of the system of the sensor. Typically a device has many sensors attached and as such we need a corresponding software element that can distinguish and route between them. Most applications are based on a single physical device with multiple sensors attached. The shadow device acts as an aggregator under an edge-computing approach, providing what appears to be a standard routing interface onto a real device element. Consequently, we have constructed the Device as a software representation of the highest-level object attached from outside the Gateway bridging points.

The Device has three basic functions.

- **Routing:** The Device is responsible for routing messages between the real sensor and the software sensor shadow. It does this by acting as an interface onto the Gateway to which it is attached.
- **Type Information Management:** All sensors, real or otherwise, are assigned a type description as a unique key defining its operation. As we presented in Section 3.2, within the framework, this key is an index to the message structure used by the sensor. Two different physical sensors from different manufactures that use the same message structure can use the same type key. The Device manages the type information, so when a new sensor appears on the Device, it negotiates directly with the sensor to retrieve the type information and construct a corresponding sensor shadow to manage its operation.
- **Device Control Functionality:** The Device itself can expose some functionality that cuts across multiple sensors. Hence there needs to be message processing functionality in the Device itself. This is done by providing a special sensor allocation, the *control sensor*, assigned the Sensor ID to the value 1. This object follows all the rules of a normal sensor and, consequently, also incorporates the ability of implementing specific functionalities locally.

All device objects can be thought of as having two sides: the first one connects to the controlling code of the shadow sensors; the second one links to the network-based servers and processing elements. In general the sensors handle most of the outgoing messages whilst the Device manages the incoming messages. When messages are directed to a Device (or any sensor on the Device) they are passed to its message processing class. The default construction of a Device object will automatically create the message processor internally. Its job is simply to route messages to the appropriate sensor and return any corresponding messages that are constructed.

A special device, known as the *Local Device* provides support for local implementation of sensors, i.e., software that conforms to a sensor interface, allowing developers to add sensors that interface directly to the gateway hardware, or use their own proprietor protocols for communications. Such a sensor as that in Listing 1 would be added to such a local device.

### 3.4. Gateway

In order to talk to real sensors, the Gateway element contains software components acting as bridging systems for the different types of connection technologies. The framework aims to be agnostic to the different underlying physical technologies and uses the Gateways to provide a standard interface onto sensors for sending and receiving messages.

**Figure 4:**
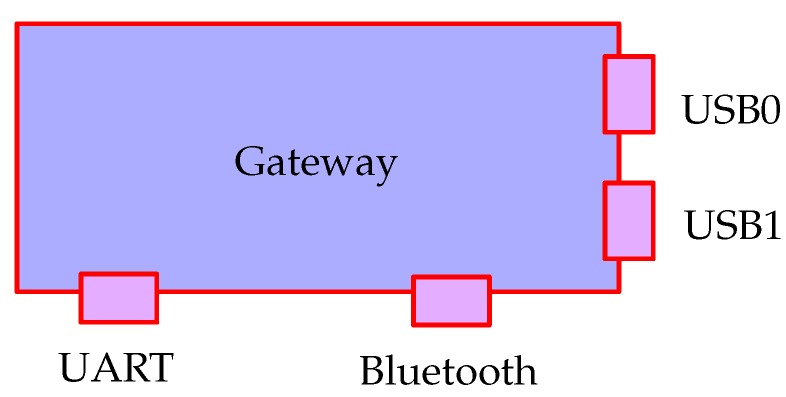
Hardware model of a gateway is a system with a collection of communication systems.

Any sensor network has at least one component in it that acts as a bridging system between the Internet Protocol (IP)-based network and the (often proprietary) sensor network system. These sensor systems and sensor networks are often connected via serial port connections or low-level hardware connections such as UART, I2C, and General Purpose Input/Output (GPIO). These connections may support one or many attached devices. The framework assumes that the bridging device is a computer capable of running a reasonably sophisticated program and it treats each available connection as a Gateway. This approach provides a general framework given that it allows treating microcontroller equipment which support IP connections as Devices connected over the IP gateway. The Gateways manage the data transfer between the network side and the Device side. Figure 4 shows an abstraction of that structure. In this case there are four active gateways attached: a standard USB (Universal Serial Bus) port, a USB port connecting a ZigBee network, a UART port, and the local BT chip. When the gateway control system is started, monitoring gateway programs need to be assigned to each of these ports. This is done by providing a gateway mapping file, which defines how each port is allocated. An example of a corresponding mapping file for the Gateway is shown in Listing 2.

**Listing 2:**
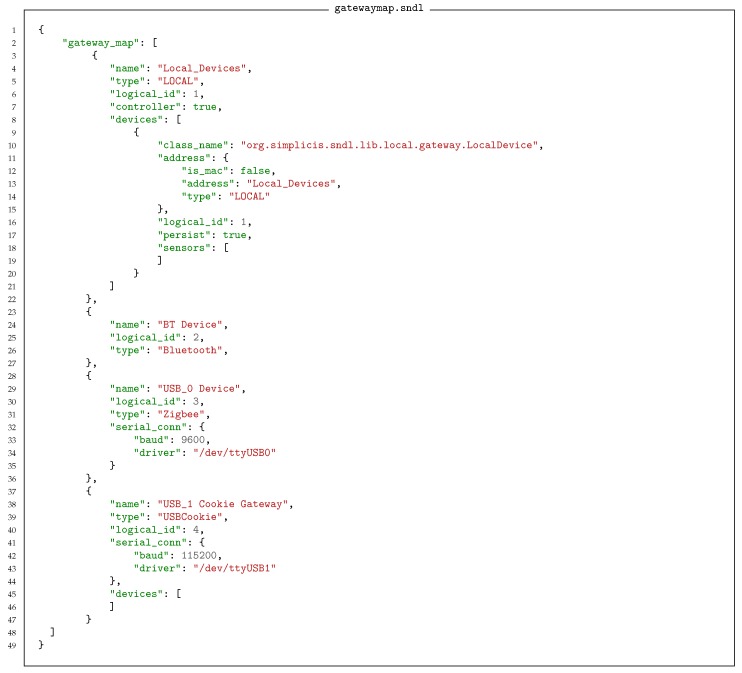
Gateway map definition. Mapping to various communications interfaces onto the correct gateway interfaces.

The configuration information is necessary in reality as there is generally no way to discover what is on each connection interface. Some systems do provide this information, but it is the exception rather than the rule. The implementation of a locally available file is needed to deal with the many errors and network blackouts a system may experience over its years of operation. It can periodically monitor for updates and changes and bring up to date its local data, but it always has to have something to fall back on if things start to fail.

### 3.5. Messaging Abstraction

The basis of our integration strategy is extremely simple and comes from the realization that messages are independent of the protocol used to represent them. Messages are simply abstract collections of data that are assigned a name allowing us to distinguish one message type from another. Specifically a message is a map that connects key names, or field identifiers, with values. This means messages are free to take different instantiated forms depending on which is the most appropriate for the given situation. It is easier to demonstrate the flexibility of message representations starting with JSON, because JSON is human readable. However, it is important to realize that our thesis is based on the fact that messages can take any representation.

**Figure 5:**
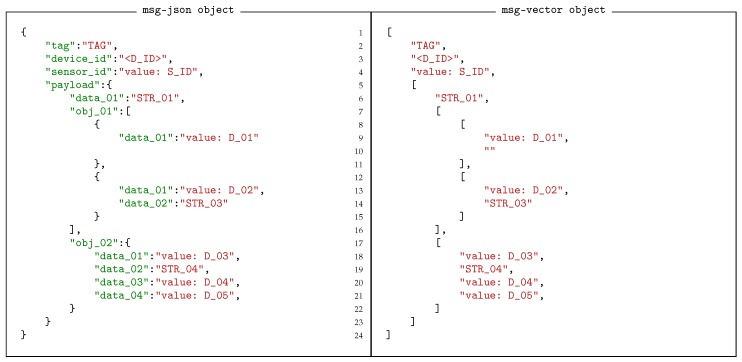
Messages reduce to maps of data, where each value can be either a native data object, or another map. However, if the format is known, the map can be replaced as a vector of values, where the maps keys are implicitly defined by their location of the vector. Here the equivalent message is show side by side as a JSON object representation and its associated vector representation.

A message can quite naturally take the form of a JSON object, where keys, or names are used to label the values. A value of course can be a native data object or another map, or vector, making it possible to have extremely complex structures. A fundamental observation is that the keys in such an object are redundant when the structure of the message is known. Hence, the message can easily be replaced with a much more compact (in general) vector form, where the position of a value implicitly defined is key. Figure 5 shows two such representations side by side. In the vector representation, missing elements must be represented by empty values to ensure each value is in the correct location of the vector. For example in Figure 5 the missing data element in the first vector object of the vector obj_01 is represented by an empty string.

**Figure 6:**
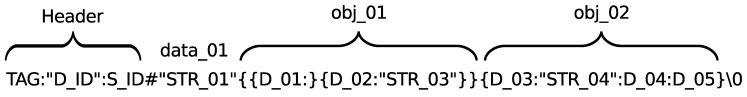
Vector message forms can be systematically serialized, where objects and vectors are delimited by curly brackets. Vectors and objects do not have to be differentiated because each value id carrying an implicate type.

Extending the concept of vectorisation, it is a simple matter to represent the message as a serial string. Each message becomes a string, terminated with a null character. Native data objects are separated with the: character. Objects and vectors are delimited by curly brackets {…}. It is not necessary to distinguish between objects and vectors as each message carries a type and therefore the type of each object is implicitly known. Figure 6 demonstrates how the message in Figure 5 transposes into a serial form. Each of the major parts are marked to indicate how they relate to the original map version of the message. The character # is used to separate the payload form the header making it easier to extract the routing information when that is all that is required.

It is also straightforward to encode the message in a variable length binary frame, using the same positioning principles. This is desirable when power efficiency and bandwidth are major concerns.

### 3.6. Modular Embedded Architecture for the Edge Devices

The sensor architecture and messaging scheme described above has been applied to several types of edge devices, but here we are focusing on the integration with the Cookie platform. This platform has been designed specifically to support the implementation of complex sensors.

In the context of the proposed complex heterogeneous sensor framework, the main target is to set a common interface definition that serves as the baseline for developers to design and include their sensor elements into the Cookie platform in a plug-and-play fashion. This means that several independent sensing capabilities can be included in the deployed sensor node through the modular architecture, and they will be properly handled by the end-to-end complex sensor management system.

The intrinsic characteristics of the Cookie platform have likewise been translated to an embedded software framework that promotes such modularity and re-usability of functional components. Following the software architecture described in Section 2.2, we have defined a library-driven and a message-based software structure on top of the embedded Cookie API [5] to give a complete support for the complex sensor platform management on the deployable nodes.

This embedded software model mirrors the representation of the shadow software architecture depicted in Figure 3. It followed naturally from the adoption of the messaging architecture and was not imposed as a requirement. It translates the functionality of the sensors directly into the embedded device and reflects the fact that the shadow sensor for such devices typically takes the form of a thin shell transporting messages across the bridge. This illustrates how the architecture naturally allows the software and hardware developers to work towards, or away from the edge depending on requirements.

**Figure 7:**
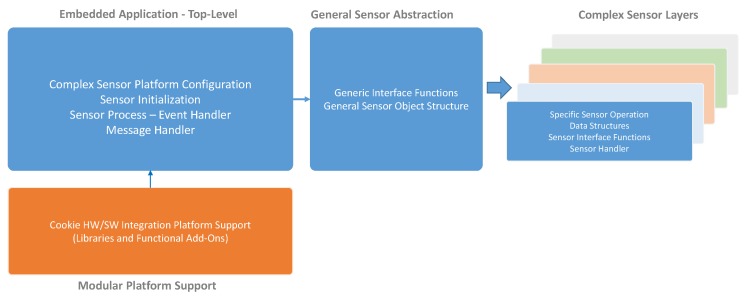
General structure of the Cookie embedded software for internally managing the integration of various complex sensors.

The overall structure of the Cookie embedded software for the complex sensor management is depicted in Figure 7. At the lowest levels we find the Software Sensor Layers, which represent modular instances of the specific definition and abstraction of the sensing technology to be used. These layers provide support for particular functionalities associated with the behavior of the hardware layer, and they may contain further libraries to process the data from the complex sensor, thus generating significant information to the upper software layers. Each layer is built from the General Sensor library which contains the underlying structure of the Complex Sensor Manager and the basic functionalities and parameters that will be provided to all the sensor layers, such as *Sensor ID*, *Sensor Type*, *Sensor State*, *Sensor Threshold*, *Sensor Last Value*, etc.

**Figure 8:**
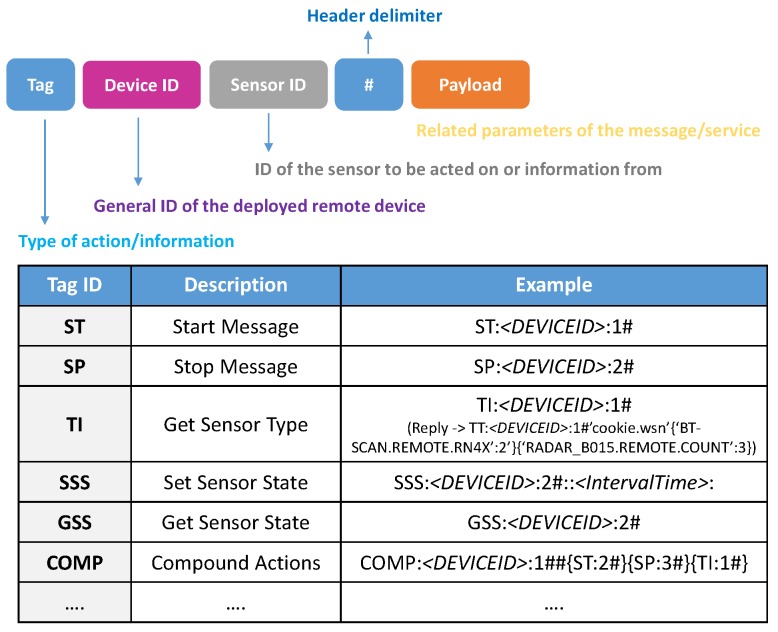
Format of the Message Structure and main actions that can be executed.

Above the sensor layers, we implement functionalities that represent the device. Specifically, the Message Handler contains the realization and processing of the incoming/outgoing packets in accordance with the Message System Architecture definition, which allows the top-level abstraction of the different complex sensor access and management. In line with the Device object definition, in Figure 8 a general view of the Message Structure is shown, which contains the various fields to properly have a bidirectional interaction with the deployed complex sensor abstraction layers on the Cookie node. In this case the representation of the messages adopts the serial form used across the serial connection to the cookies WSN and not the form used in the radio packets, or the JSON form more typically used by application developers. Figure 8 illustrates the basic actions that can be performed remotely to monitor or configure/update the operation a Sensor/Device. Such a definition allows discriminating the element(s) upon which a configuration or information retrieving will be performed. A configuration, data or property retrieval can be carried out at three levels:

- Device level: It refers to intrinsic configuration or information of the Cookie node, and it provides support for gathering information from or sending configuration to all the plugged sensor layers in the same message stream. The device is effectively addressed by setting Sensor_ID=1.
- Sensor level: Specifically, parameters, configuration, and data gathering from a particular sensor associated with a specific sensor level.
- Composite level: This allows the multiplexing of multiple messages from various sensors into a single message body. In effect it eliminates the need to duplicate the Device_ID when many messages are being generated by that device.

At this point it is important to note that the message representations are optimized for operation at specific points in the information flow pathway, i.e., the different physical systems the message passes through as it traverses the network. At the edge point we are considering, the messages are flowing across a serial channel, so we use that representation of the message which is more suited to the hardware developer.

## 4. Real Use Case: Bluetooth Identifier and Radar Counter

Following the proposed development framework we have built an integrated traffic sensor that combines a radar counter and a BT identifier to provide complementary traffic information.

### 4.1. Radar Counter

The radar sensor used in our integrated platform has been implemented on a single Printed Circuit Board (PCB) following the Cookie architecture. Radar signals are generated and received by the radar transceiver K-LC5 from RF beam [23]. It is a low-cost wide-beam transceiver which continuously generates 24.150 GHz signals when powered-on, and provides the mixed I and Q signals returned. The amplitude of these signals is small, so an amplifier stage is used in our board to amplify them. In addition, an anti-aliasing filter and a single-ended to differential converter are implemented to adapt the I and Q signals before digitalizing them.

The integrated analog to digital converters (ADCs) included in the STM32L496 microcontroller are used to digitize the returned signals. These 12-bit ADCs perform simultaneous acquisition mode to sample I and Q signals. In addition, the oversampling hardware engine is used to increase the effective resolution to 16 bits. The acquired samples are then stored in the microcontroller memory by using direct memory access (DMA). The microcontroller used runs up to 80 MHz of clock frequency and includes a floating point unit which allows using it to process the stored samples and provide counting data. These generated data are sent to the processing layer when they are requested using a slave SPI interface.

Finally, the low-noise requirements of the radar hardware make it necessary to implement an independent power supply module instead of using the Cookie power supply framework. This module provides independent supply voltages for the analog and digital modules. The radar transceiver, the amplification and filtering modules are powered by the analog supply which provides enough current for them with small ripple, as high ripple values reduce acquisition performance.

### 4.2. Bluetooth Identifier

The variation on the BT scanner designed for this type of deployment uses a standard BT module, the Microchip RN41 [24], which features a low-power certified Class 1 BT Radio with on-board embedded stack, and with a UART interface. This has proved to have sufficient processing speed to deal with scanning for vehicles moving with a high velocity. Similar to the radar-based sensor layer, the BT identifier has been designed following the modular architecture of the Cookie presented in previous sections.

Seeking the desired plug-and-play capability a small dedicated program converts the propitiatory serial protocol into the message structure for a location scanner. In this case the system transparently sees the Cookie as a device regardless as to whether the Cookie is connected as a serial device, or via the sensor network.

### 4.3. Information Flow within the Hardware

**Figure 9:**
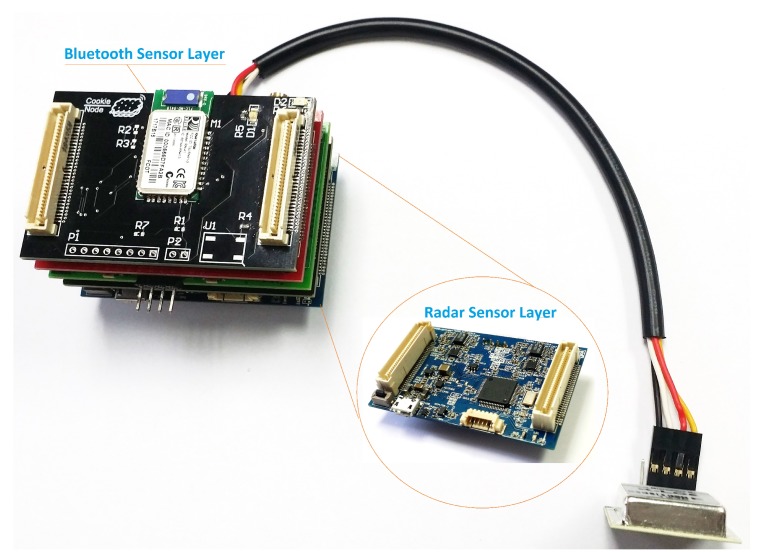
Hardware integration result of BT + Radar complex Sensors into the Cookie platform.

The result of the Cookie complex sensor integration is shown in Figure 9, where the Sensor node comprises the two designed layers plus the power supply, processing based on an 8-bit 8051-based low-power ADuC841 microcontroller from Analog Devices (Norwood, MA, USA) and WSN wireless communication (which includes a IEEE 802.15.4 compliant CC2420 radio chip from Texas Instruments) modular layers. It is important to highlight at this point that the combination of the modular features of the Cookie platform with the characteristics of the wireless networked system allows a straightforward modification of the deployment topology in accordance with the requirements of the target area. It means that one Cooke node can be composed of both complex sensors and placed in a strategic point of the traffic zone to be monitored, and the network can be diversified with one additional node with only BT scanner for data filtering and redundancy checking, and so on.

In the context of this real use case, and considering the proposed message structure, the BT scanner sensor is identified by *Sensor ID = 2* whereas the radar sensor has *Sensor ID = 3* (The Cookie node acting as the Device has the *Sensor ID = 1*). Table 1 summarizes the main properties and configurations available for these implemented complex sensors that can be remotely accessed, so as to dynamically modify or update the behavior of the deployed sensors.

The basic operation of the radar sensor is defined by capturing the number of detected vehicles in a configurable period of time and the associated speed they go through the detection zone. In the predefined configuration, the Sensor Handler of the Cookie Node process the value of the card detection and speed array every 1 s. The result of the speed array will be transmitted to the base-station node (and thus to the Gateway) whenever a number of cars within the sample window is higher than 0. In the meantime, the BT sensor will operate in the detection of MAC identifiers by using the inquiry state configuration, which scenically opens a scanning window to capture the BT MAC address of the vehicles in the surrounding area. The Cookie node will automatically deliver the state of the inquiry by providing the gateway with a valid recognized MAC ID through the corresponding message transmission frame.

### 4.4. Integrated Vehicle Counting and Identification Data

The developed integrated platform is capable of producing both types of data (vehicle counting and identification), in a standardized way that eases the data fusion to be performed on the higher processing layers.

Illustrative examples of real data generated by BT vehicle identifiers and radar vehicle counters are shown in Listings 3 and 4 respectively, both in JSON and serial formats. Looking at the payload fields of the data produced, we can observe how BT sensors provide information about the time it detected the vehicle, the MAC address and the DIAC (*profile*) of its embedded BT device, and the received signal strength indicator (RSSI). Accordingly, radar counters supply information containing a time stamp and the set of velocities of each detected vehicle.

**Listing 3:**
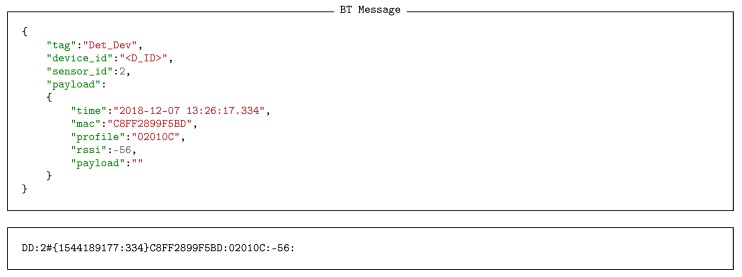
BT detection message in JSON and serial format.

**Listing 4:**
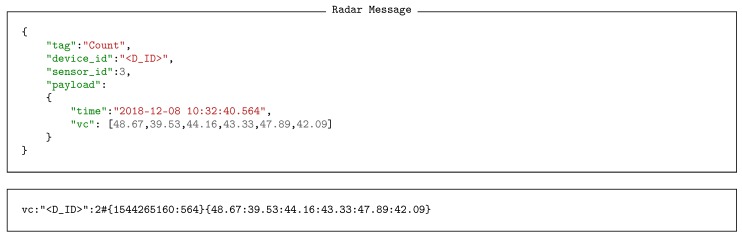
Radar detection message in JSON and serial format.

## 5. Results

### 5.1. Data Rate Reduction of Complex Sensors

The complex radar sensor used in our platform is continuously sampling the I and Q signals returned by the radar transceiver. The sampling frequency has been set to 12.5 KHz, while the resolution of the ADC is 16 bits (with oversampling). Therefore, we are generating a continuous stream of data of 25 KB/s for each I and Q channel. The sum of both streams implies a data rate of 50 KB/s. As we use DMA in the radar microcontroller, the data is continuously sampled while previous stored data can be processed by the CPU. The processing window has been set to 128 samples, so this amount of data is sampled while the ADCs are acquiring the next 128 samples.

Acquiring 128 samples at 12.5 KHz of sample rate takes 10.24 ms, while the signals processing time has been measured and is lower to 9.5 ms. Acquiring and processing the signals without overrunning is therefore feasible as the processing time is lower to the acquisition time. Therefore, when 128 samples have been processed velocity data is obtained each 10.24 ms. As velocity data is represented with a floating point 32-bit number, and assuming detecting one moving target, we are generating 390.625 B/s. This processed velocity data stream is much smaller than the sampled 50 KB/s data stream. Therefore, we summarize in Table 2 this experimental set up for the radar sensor.

Then, we assume a radar range of 10 meters and a vehicle speed of 120 km/h, we may count up to 3 vehicles per second in a real-world deployment. We represent the data counting of each vehicle by its associated floating-point 32-bit velocity, so only 12 B/s are generated in this case. As observed, by processing radar data on the edge we have reduced the large amount of data sampled from the radar transceiver. The 50 KB/s data stream is reduced to a maximum of 12 B/s of meaningful data. Table 3 summarizes this data reduction in our edge-computing radar sensor. It is important to highlight that we have presented the worst-case data rate results obtained when the radar sensor is continuously detecting 3 vehicles per second, which is not the most common scenario. In a more realistic scenario, the sensor may be inactive (not detecting vehicles) during long periods of time, thus generating no output data. So in this case, the output data rate is reduced to zero from the original 50 KB/s data stream, which is the desired target for any edge-computing platform.

Finally, the vehicle counting data generated by the radar sensor is transmitted through a slave SPI interface to the processing layer. A fixed-sized packet of 44 bytes is sent by the radar sensor when the integration platform requests it. It contains the vehicle counting value in a 32-bit integer format, and up to 10 velocities of each vehicle counted in a 32-bit floating point format.

On the other side, raw data generated by the BT identifiers mainly include the vehicle identifier and the time stamp of the detection. Our previous deployments of BT detections systems have specifically used cloud-based processing to retrieve these raw data and extract relevant traffic information. Typically what is needed in traffic management systems is not the retrieved location data, but the travel time of a monitored stretch, defined between two designated sensors. However, this application is ideally suited to migration towards the edge of the network. By defining a virtual sensor that consumes the data generated by the far edge sensors, specific point to point travel time calculations can be performed on the local gateway.

Travel time is calculated for each link, defined by a pair of nodes (origin and destination). These two nodes perform a significant amount of detections every day. However, just a minimal percentage of these detections can be actually used to calculate travel time in a specific link. First, in order to produce an accurate measurement, the travel time calculation must discard any detection that comes from devices that do not precisely correspond to a vehicle. Thus the first filter separates hands-free devices from mobile phones, laptops, etc. Second, only vehicles that are detected by sensors in both the origin and destination of the link really contribute to a travel time estimation; for example, vehicles detected by a sensor, which are circulating on the opposite direction to the destination are obviously not included in the calculation. Third, among the set of vehicles detected at both ends we can also find *outliers*, i.e., vehicles that did not travel the stretch at a coherent speed compared to the others; consider for example a commuter that was only detected at the origin in the morning and only detected at the destination in the afternoon. Last, we need to collect certain number of detections to provide a statistically reliable calculation of travel time; we have currently fixed this value to 15. Table 4 shows an illustrative example of the data reduction achieved step-by-step by using a virtual sensor that follows the sequence of filtering and calculations to provide travel time measurements. The data was captured on 6 November 2017, and correspond to a stretch of José Abascal street, from Bravo Murillo street to Gregorio Marañón square, in the city of Madrid (Spain).

According to these results, a large amount of data that is consumed locally can be discarded, effectively compressing the data stream. The virtual sensor is literally regarded as a different type of sensor providing travel time information, so that other processing modules can consume this data as an ordinary input.

In this case, inadvertently, there is also an improvement in security and privacy, as tracking data can be eliminated before there is any chance it can be intercepted. It is interesting to note this is an example of how the architecture of the system can be evolved and extended after deployment without the need to modify/upgrade any of the deployed technology. It is simply a matter of adding a new sensor definition, i.e., software deployment at the edge of the network.

### 5.2. Integration of Complex Sensors

The proposed platform offers a whole set of options to integrate sensors, which is key for the real fusion of complex sensors data.

The first alternative is to treat the complex sensor as an independent piece of equipment providing already processed data to the platform. In this case, the complex sensor will include its own hardware and software processing system and will connect to the platform through the corresponding sensor shadow as we described in Section 3.2.

A second alternative allocates the first-level processing required by a complex sensor to produce significant data in a specific Message Handler attached to a Sensor Body. The input of raw data will be passed to this processing element from the real sensor, acting as a serial device connected to the system.

Finally, a third possibility of data fusion is to assign the processing capabilities to the Device and let this piece of code be responsible for extracting the first meaningful information from the complex sensor. This approach could be especially beneficial for those applications where data provided from a set of complex sensors must be fused to obtain the desired output; for instance, think of a radar that provides distance information of the detected objects that has to be integrated to generate their exact geographical position.

Placing the first-level processing capabilities required by complex sensors on the sensor shadow (Message Handler) or the Device will not reduce the overall performance of the system as long as there exists a high capacity communications channel between the real sensor and the computational device where the software processing elements are run. Most complex sensors such as cameras, radars, environmental sensors, etc., produce high data loads to be processed. If the real sensors are connected to the system through a local IP channel, or direct cable connection, the high data load they generate will not be an issue. However, we must bear in mind that the code instantiating the Sensor Body or the Device could be allocated anywhere in the network and not restricted to physical devices of the form of SBCs or aggregators that are physically connected to the real sensors. In this case, the system performance could dramatically drop, or incur in large operating costs, due to the communications channel. Consequently, the design of the overall system has to take into consideration this fact in order to avoid a potential deterioration of the system’s performance.

### 5.3. Data Fusion of Complex Sensors

The integrated vehicle sensor provides the radar-based counting and BT-based identification data presented in Section 4.4. The fusion of these two types of data is key to traffic engineers as it extends the individual knowledge obtained separately from each class of sensor.

As an illustrative example of this extended information, we can fuse travel time measurements and vehicle counting produced by the integrated traffic sensor, to obtain *weighted* values of travel time. Travel time on its own indicates the time employed by vehicles to cover a specific stretch in a certain moment during the day; however, it cannot inform about how many vehicles did actually travel that distance. On the other hand, radar counters can report the number of vehicles per unit time that have gone through a specific point on the road network; nevertheless, they cannot state how many of these ultimately reached the destination or made some turn before arriving.

By combing data from multiple sources, weighted traffic time values can be constructed. Such measurements enhance and extend the raw information present in the contributing sensors and create more significant and digestible information. For example, in this case, not only does the data provide meaningful information about the estimated transit time, it includes the number of vehicles that can be expected to reach the destination in the specified time interval. Consequently, traffic engineers can take informed decisions to manage the operation of traffic lights to reduce potential congestions, or just as importantly more sophisticated monitoring and data processing modules can be constructed basing their decisions on this extended information. Listing 5 shows an example of a weighted travel time in both JSON and serial formats. In this case, the payloads include the minute of the day and link corresponding to the measurement, the time interval over which the calculation was made, the travel time value and its confidence interval, and the number of vehicles that started the journey on the origin node.

In general it is likely that the processing element, or virtual sensor making such calculations is a local sensor running inside a server in the network and hence, the JSON representation show more useful because of the local availability of high bandwidth communications and the likelihood that end applications will be directly interested in the generated data.

**Listing 5:**
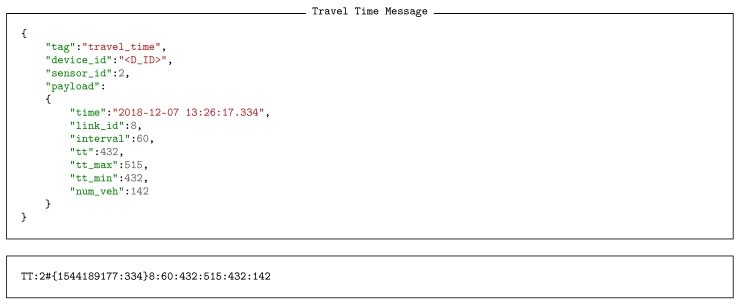
Weighted travel time message in JSON and serial format.

### 5.4. Optimization of the Network Overhead for the Data Fusion

**Figure 10:**
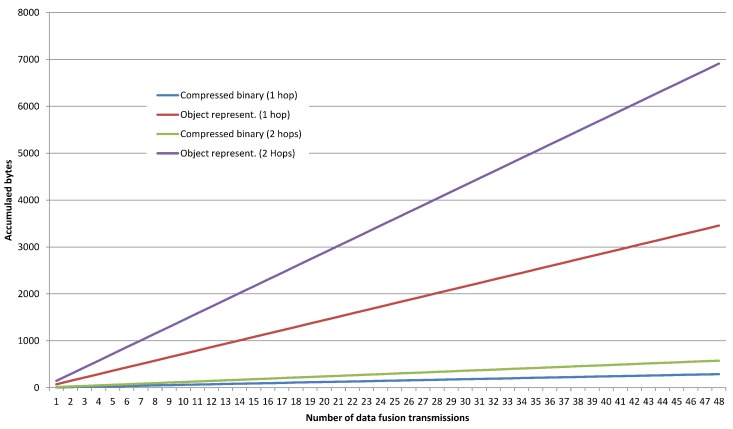
Comparison of network overhead for two different types of data fusion transmission formats.

Figure 10 shows a comparison of the traffic overhead on the IoT Edge when applying an optimized compressed binary format for the data fusion of complex sensors and the serialized object representation. It encounters the transmission of 50 packets from the Sensor Node to the Gateway using one single hop and two hops. I can be clearly seen that as the depth of the network increases (number of hops from the edge sensors to the gateway) the difference between the compressed binary format dramatically reduced the traffic overhead compared to the normal transmission (91.67% of optimization), so the long-term network lifetime will be indeed enhanced with that additional enhancement.

### 5.5. Reduction of Development Times

The integration of new complex sensors by means of the proposed end-to-end architecture, and particularly at hardware level by using the Cookie modular platform, allowed reducing development time of each multidisciplinary team, highlighting three main aspects:

- **Independent hardware design and implementation:** The modularity of the Cookie architecture accelerated the effective inclusion of the two complex sensors for this real use case. This was a result of having design rules to specify and implement a new Cookie layer within the architecture, and parametrized hardware interfaces in the form of hardware design templates available for the developers. This fact does not only encompass the integration of a particular sensor with a fixed processing element (as is the usual case in commercial devices), but also relying on trivial re-usability of a large family of pre-existing Cookie layer IoT hardware technologies (the result of 10 years of applying this architecture in several real deployments.) This includes: Various processing technologies (a combination of different microcontrollers and if more intensive processing is required FPGAs are also available), wireless communication (including wide-extended low-power wireless network protocols,) and flexible power supply. Users do not need to perform the design of a new overall board to integrate their new complex sensor, but just a new Cookie layer that can be directly plugged into the modular stack. If they need particular requirements for the target application, they can evaluate the repository of hardware layers and, in case a unique feature is necessary to comply with specific constraints and they are not covered by the repository, a new layer can be designed without affecting the other layers (for instance, the processing layer can be empower with a desired processing specification but the communication technology will remain unaltered). Table 5 summarizes the development time reduction based on authors’ experience in the hardware design for this real use case.
- **Manufacturing Costs:** Based on the aforementioned modularity and re-usability of the Cookie architecture, the manufacturing of the network prototype is considerably reduced in terms of complexity and cost, since the process is indeed focused only on those independent layers rather than the (re)design of the overall embedded platform to be deployed. Specifically, according to the real expenses of the proposed complex sensors, there is a 90% of cost reduction in the manufacturing process, which was also reflected in a shorter delivery time for the final prototypes.
- **Integration effort at both hardware and software levels:** The modularization of the software and the standardized messaging approach has a similar effect on incorporating a new sensor into the network side. The modularized library of the Cookie makes it much easier to add messages to the input/output of the Cookie module.
  In addition the standard form of the messaging means the gateway side of the software needs little, or no modification. Unless message representation conversion is required in the gateway nothing needs to be added. A new sensor board can be implemented, then simply turned on and its messaging will appear in the network. This does not mean to say that the processing of the messages does not need to be designed and implemented (which is simply an application level process), but the mechanism of getting the information from the sensor to wherever it is required is already available.
  This significantly simplifies the joint development of hardware and software teams, which only have to concentrate on their own requirements letting the common messaging interface deal with the interaction between these two areas.

## 6. Conclusions

In this work, we have presented an edge and fog computing platform which facilitates the integration of complex heterogeneous sensors within a common management framework. The main contribution is the symbiotic hardware/software design approach. It is achieved by treating the sensors as services in a SOA and using a messaging abstraction that provides common functionality that can be tailored to each specific requirement in the communications path.

Sensor services can identify themselves if they are real physical systems, or can be defined by a standard JSON representation which defines the operation of each virtual, or locally configured sensor. Regardless of their origin, sensors are defined by the messages they produce and consume making them a standard element within the framework. Therefore, the definition of a sensor provides an efficient and well-established interface, making it trivial to design a virtual sensor, providing a common ground for heterogeneous data sources.

The SOA and abstract messaging system has been embedded in a modular hardware platform based on a layered architecture which provides a set of digital, analog and power-supplying interfaces. Heterogeneous physical sensors may be designed for each application in compliance with this hardware architecture, while the messaging structure allows their top-level integration into the overall system by just defining the associated complex sensor messages.

To demonstrate the ease with which this approach facilitates coordinated design and development activity across the software and hardware teams in collaborative projects a real use case is presented. Here we show the suitability of our integration platform in a real-world traffic monitoring scenario. In that context, a radar counting sensor and a BT vehicle identification sensor are implemented and fully integrated into the modular platform. Both are considered as complex sensors and are developed as independent hardware layers compliant with the Cookie architecture. The messaging system has provided the developers with the framework required for the management and data fusion of the radar and BT sensors, so that the operation of the traffic monitoring system could be achieved.

Our empirical performance metrics show improvements in the efficiency of the system itself and the development time. In particular, the use of the proposed framework has reduced the data rate produced by the radar counter and the BT identifier in three orders of magnitude. In addition, we open up to three possibilities for the integration of complex sensors into new or already existing systems. Once the data is collected, our framework provides the means to fuse them, creating new virtual sensors, regarded as one more type of information source that can be consumed by higher processing layers. This way, the vehicle counting and the calculated travel time are fused to generate a novel weighted travel time, relevant for traffic engineering purposes. Finally, apart from this quantifiable improvements, our framework provides intangible gains allowing developers on both hardware and software sides to operate in parallel. The messaging definition of the sensors allows the software engineers to use a model of the sensor operation before it physically exists. Correspondingly, the hardware engineers are insulated from requirement changes at the level of the application as the messaging definitions are remarkably stable throughout the development process.

In the future, the proposed integration platform will be used in diverse application use cases to benefit from such a modularity during the integration of new complex sensors. In the short term, several deployments of the traffic monitoring system will be carried out to further analyse the data fusion capabilities combining different network topology strategies for the complex sensors.

## Figures and Tables

**Table 1:** Main configurations available in the integrated complex sensors.

Type of Sensor	Parameter	Action
Bluetooth scanner	Inquiry Window	Configures the maximum time of MAC scanning per inquiry.
	Inquiry Trigger Interval	Configures the timeout after with a new Inquiry is to be triggered.
	Transmission Power	Configures the value of the transmission power in dBm.
	Detection Delay	Configures the delay between two detections.
Radar traffic counter	Vehicle Direction	Configures the type of vehicle detection in accordance with the sensor orientation (Vehicle approaching/exiting).
	Noise Threshold	Tunes the noise level threshold to adapt the radar to the particular conditions of the scenario.
	Led Notification	Configures the behavior of the Onboard Led (for Debugging purposes).

**Table 2:** Experimental setup for the counting radar sensor.

Parameter	Experimental Setup
Sample rate	2 × 12.5 KB/s
Window Size	128 samples
ADC resolution	16 bits
Velocity type	32-bit float
Radar range	10 m

**Table 3:** Data rate reduction from radar sensor vehicle counting.

Data	Data Rate (KB/s)	Accumulated Data Reduction
Unprocessed Data Stream	50	-
Velocity Data (1 target)	0.390	128/1
Counting Data (3 vehicles each second)	0.012	4167/1

**Table 4:** Data rate reduction on travel time estimation from BT vehicle identifiers.

Data	Number of Records	Accumulated Data Reduction
Single detections in origin and destination	68,759	-
Double detections in the corresponding link	2849	24.13/1
Valid double detections (discarding outliers)	1919	35.83/1
Calculated travel times	212	324.33/1

**Table 5:** Comparison of the development time for the proposed complex sensors.

Complex Sensor Layer	Normal Development + Testing Time Estimation (Person/Hour)	Framework-Based Development + Testing Time (Person/Hour)	Reduction (%)
Bluetooth board	140	35	75
Radar Board	350	70	80
